# Experiences of postnatal contraceptive care during the COVID-19 pandemic: a multimethods cross-sectional study

**DOI:** 10.1136/bmjopen-2024-095608

**Published:** 2025-06-17

**Authors:** Shauna Kelly, Malcolm Moffat, Caitlin Thompson, Robyn Jackowich, Christine Möller-Christensen, Claire Sullivan, Judith Rankin

**Affiliations:** 1 Newcastle University, Newcastle upon Tyne, UK; 2 Cardiff University, Cardiff, UK; 3 Gateshead Health NHS Foundation Trust, Gateshead, UK; 4 Office for Health Improvement and Disparities, London, UK

**Keywords:** COVID-19, Pregnancy, Postpartum Period, SEXUAL MEDICINE

## Abstract

**Abstract:**

**Objectives:**

This study aimed to examine the impact of the first COVID-19 lockdown period on access to postnatal contraception (PNC) and wider postnatal care and to explore the experiences of PNC care within the North East and North Cumbria (NENC) Integrated Care System (ICS) during the same period.

**Design:**

This study reports a subanalysis of the NENC Postnatal Contraception (PoCo) study, an online survey of a convenience sample of women in the NENC ICS who completed pregnancies between 2019 and 2023.

**Setting:**

Women who completed pregnancies between 2019 and 2023 in the NENC ICS.

**Participants:**

Out of the total 2509 eligible participants who completed the PoCo survey, women who delivered in April–June 2020, April–June 2021 and April–June 2022 were included within this subanalysis, resulting in 457 eligible survey responses. There were no additional exclusion criteria.

**Primary and secondary outcome measures:**

Primary outcome measures were PNC uptake and number of healthcare professional contacts during the postnatal period. Secondary outcome measures were self-reported experiences of PNC care.

**Results:**

Women who delivered in April–June 2020 had fewer postnatal contacts than women who delivered in subsequent non-lockdown cohorts and were less likely to be offered PNC prior to discharge. There were no significant differences in relation to PNC uptake. In qualitative analyses, several women who delivered in 2020 highlighted COVID-19 as a factor perceived to be associated with poor postnatal care. Across all three groups, experiences of PNC care were diverse; feeling pressured to accept PNC was frequently reported.

**Conclusions:**

While the first COVID-19 lockdown appears to have had a significant impact on women’s experiences of postnatal care, this did not result in a substantive decrease in PNC provision, likely reflecting pre-existing shortcomings. These women and families may benefit from additional support postpandemic to mitigate the potential life course implications of restricted support in the postpartum period, and policy-makers and healthcare providers should continue to explore innovative and patient-centred approaches to improving PNC provision. Future research should continue to evaluate the longer-term impacts of these changes in non-pandemic contexts.

**STRENGTHS AND LIMITATIONS OF THIS STUDY**

- Uses quantitative and qualitative methods to respond to the research question.
- Captures the demographic diversity of the sample population in relation to some, but not all, characteristics.
- Uses a relatively small sample size for some comparisons/analyses, which may have resulted in reduced statistical power/increased likelihood of type 2 errors.
- Possibility for recall bias/error, with participants asked to describe events that occurred several years earlier. The group of participants that completed their pregnancies in 2020 may have been at highest risk of recall bias.
- The absence of a pre-COVID-19 participant cohort precluded the possibility of comparisons with prepandemic experiences/outcomes, which would have been valuable.

## Introduction

 Access to contraception is a human right, and effective family planning care is essential in reducing maternal morbidity, pregnancy-related deaths, unsafe abortions and in improving opportunities for women in work and education.^1^ Postnatal contraception (PNC) care benefits both maternal and infant health, through prevention of short interpregnancy intervals that have been linked to preterm birth, low birth weight and infant mortality.^2^ Prior to COVID-19, it had been recognised that postnatal care in the UK required improvement.^3^ However, it has been suggested that the public health response to COVID-19 in the UK was often suboptimal, and this may have further perpetuated challenges in postnatal care and PNC provision.^4^

Women who completed pregnancies in the first UK national lockdown did so during a period when people were ordered to stay at home and only leave for essential purposes, such as to access medical care.^5^ With pregnant women identified as especially vulnerable, they were advised to avoid all unnecessary social contact.^6^ Changes to care provision during this time included the use of ‘smart devices’ for antenatal and postnatal care, centralisation of care to obstetric-led settings, and restrictions on partner and family visiting.^7^ The UK roadmap out of lockdown, published in February 2021, detailed the gradual lifting of social contact restrictions in line with protecting the National Health Service (NHS), beginning on 29 March 2021 and with most legal restrictions eased by 19 July 2021.^5 8^ The 24 February 2022 marked the end of all legal pandemic restrictions, with women who completed pregnancies after this point being unaffected by COVID-19 restrictions.^9^ The existing academic literature refers to opportunities presented by COVID-19 to develop novel and innovative approaches to PNC provision in the UK, in light of policy changes such as recommendations that midwives should be permitted to prescribe the progestogen-only pill in maternity settings.^10 11^ Studies show that these initiatives, as supported by temporary ‘emergency’ funding and staff redistribution, were received positively, especially with regard to ensuring that high-risk women received adequate PNC care.

The North East and North Cumbria (NENC) Integrated Care System (ICS) is the largest ICS in England, serving a population of just over three million people. With an annual birth population of 25 000, it has the highest rate of conceptions in individuals under 18 in England, and one of the highest postnatal abortion rates in individuals under 25.^12 13^ This subanalysis uses data collected in the NENC Postnatal Contraception (PoCo) study, an online survey that examined women’s experiences of antenatal, intrapartum and postnatal care in general and their experience of PNC care in particular.^14^ The survey collected data from women who completed a pregnancy in the NENC ICS between 2019 and 2023 and included closed and open (free-text) questions. Headline findings have reported that PNC uptake was low in this sample and that uptake varied by a number of demographic and pregnancy-related characteristics. While previous studies have investigated pregnancy outcomes following antenatal infection with COVID-19, none have examined the impact of COVID-19 on PNC care in particular.^15 16^

The aim of this study was to examine the impact of the first COVID-19 lockdown period on access to PNC and wider postnatal care and to explore the experiences of PNC care within the NENC ICS during the same period.

## Methods

The PoCo study was an online survey of women who completed a pregnancy between 2019 and 2023 in the NENC ICS, recruited through convenience sampling between 1 December 2022 and 3 April 2023 by means of social media posts, recruitment in healthcare settings (hospitals and general practitioner (GP) practices) and engagement with support groups.^14^ The survey was developed in collaboration with a patient and public involvement (PPI) group and a project steering group, and the final survey was piloted by both groups. The present study, a subanalysis of the PoCo study, explored the experiences of women who delivered during the first COVID-19 lockdown (April–June 2020 group) as compared with those who delivered during the equivalent periods in subsequent years (April–June 2021 group and April–June 2022 group, total eligible sample 457 respondents). Responses from these three groups of women were examined and compared, using quantitative and qualitative methods to explore the impact of COVID-19 on uptake of PNC, on access to PNC care and wider postnatal care and support more generally, and on self-reported experiences of PNC care. Detailed information about the methods and survey design is available in Moffat *et al.*^14^ A PDF copy of the survey is available as online supplemental Appendix 1. Respondents were eligible to participate and to be included in this sub-analysis if they completed a pregnancy during one of the three previously specified time periods in the NENC ICS. There were no additional exclusion criteria.

Statistical analyses were undertaken using SPSS V.27 (IBM). Descriptive statistics were generated, and the χ^2^ test of association was used to examine associations across the 3-year groups (April–June 2020, 2021 and 2022) in relation to participant demographics, pregnancy-related characteristics and experiences of PNC care, with statistical significance set at p<0.05. In logistic regression analyses, odds ratios (ORs) with 95% confidence intervals (CIs) were estimated to examine any differences between the three cohorts in relation to uptake of PNC, by grouped (any medically prescribed or administered PNC type and any LARC method) and individual contraception methods. CIs that did not cross 1 were considered statistically significant (p<0.05).

Free-text questions were included in the survey to allow respondents to describe their experiences of PNC care and to share suggestions as to what was done well and what might require improvement. These responses were extracted from the data and uploaded to NVivo for coding and thematic analysis.^17^ Three reviewers (SK, CT and MM) independently reviewed the responses and generated codes inductively, which were used to create a shared codebook. 20% of responses were dual coded to confirm the reliability of the codebook. Themes based on the codes were generated and agreed on by SK, CT and MM.

### Patient and public involvement

The PoCo study was designed by a multidisciplinary steering group with support from a PPI panel. PPI panel members were women resident in the NENC ICS with recent lived experience of pregnancy and postnatal care. The panel advised on the development of the research funding proposal, on the writing of the study protocol and documents, including the survey questions, and the interpretation of results was discussed with the group after the study was launched. The PPI group also supported the researchers with suggestions for study recruitment.

## Results

### Participant demographics

A total of 2509 eligible participants completed the PoCo survey during the full study period (2019–2023). The present substudy extracted 457 eligible responses from those participants who delivered in April–June 2020 (19.2%, n=91), April–June 2021 (32.6%, n=149) and April–June 2022 (47.5%, n=217). Missing data accounted for <10% of responses for all variables.

Demographic characteristics are described in table 1. Most respondents were aged 25–34 (74.6%, n=338), heterosexual (95.6%, n=434) and white British (97.6%, n=446). Overall, 26.7% (n=106) of respondents lived in postcodes in Index of Multiple Deprivation quintile 1, representing the 20% most deprived postcode areas in England.

**Table 1:** Demographic and pregnancy-related characteristics of respondents

			Year, deliveries occurring between April and June				
**Demographic- and pregnancy-related characteristics**		**Total sample**	**2020n (%)**	**2021n (%)**	**2022n (%)**	**χ2 (d.f.)**	**P value**
Age	<19 years	10 (2.2)	2 (2.2)	5 (3.4)	3 (1.4)	8.56 (10)	0.57
	20–24 years	54 (11.9)	7 (7.8)	17 (11.4)	30 (14.0)		
	25–29 years	166 (36.6)	31 (34.4)	60 (40.3)	75 (35.0)		
	30–34 years	172 (38.0)	35 (38.9)	51 (34.2)	86 (40.2)		
	35–39 years	47 (10.4)	14 (15.6)	15 (10.1)	18 (8.4)		
	>40 years	4 (0.9)	1 (1.1)	1 (0.7)	2 (0.9)		
Sexual orientation	Straight (heterosexual)	434 (95.6)	89 (97.8)	141 (95.3)	204 (94.9)	1.35 (2)	0.51
	Other	20 (4.4)	2 (2.2)	7 (4.7)	11 (5.1)		
Relationship Status	Married	239 (53.0)	55 (61.1)	80 (55.6)	104 (47.9)	**14.20 (6)**	**0.03**
	Civil partnership	16 (3.5)	3 (3.3)	4 (2.8)	9 (4.1)		
	Relationship	173 (38.4)	24 (26.7)	51 (35.4)	98 (45.2)		
	Single	23 (5.1)	8 (8.9)	9 (6.3)	6 (2.8)		
Ethnicity	White UK and Irish	446 (97.6)	90 (98.%)	145 (97.3)	211 (97.2)	0.83 (2)	0.66
	Any other ethnicity	11 (2.4)	1 (1.1)	4 (2.7)	6 (2.8)		
Postcode IMD quintile	1	106 (26.7)	21 (26.9)	37 (27.6)	48 (25.9)	3.41 (8)	0.91
	2	93 (23.4)	18 (23.1)	34 (25.4)	41 (22.2)		
	3	59 (14.9)	14 (17.9)	19 (14.2)	26 (14.1)		
	4	82 (20.7)	17 (21.8)	27 (20.1)	38 (20.5)		
	5	57 (14.4)	8 (10.3)	17 (12.7)	32 (17.3)		
Household income	£39 000 or less	180 (40.6)	33 (38.4)	61 (42.4)	86 (40.4)	0.59 (4)	0.96
	£40 000–£69 000	175 (39.5)	34 (39.5)	55 (38.2)	86 (40.4)		
	£70 000 and above	88 (19.9)	19 (22.1)	28 (19.4)	41 (19.2)		
Education level	Level 1 and no qualifications	24 (5.3)	5 (5.5)	8 (5.4)	11 (5.1)	1.03 (6)	0.99
	Level 2	67 (14.7)	11 (12.1)	22 (14.8)	34 (15.7)		
	Level 3	120 (26.3)	24 (26.4)	37 (24.8)	59 (27.2)		
	Level 4 and above	246 (53.8)	51 (56.0)	82 (55.0)	113 (52.1)		
Self-reported mental health	Very good	75 (16.4)	10 (11.0)	22 (14.8)	43 (19.8)	7.54 (8)	0.48
	Good	226 (49.5)	48 (52.7)	74 (49.7)	104 (47.9)		
	Fair	125 (27.4)	29 (31.9)	40 (26.8)	56 (25.8)		
	Bad	30 (6.6)	4 (4.4)	13 (8.7)	13 (6.0)		
	Very bad	1 (0.2)	0 (0.0)	0 (0.0)	1 (0.5)		
Self-reported physical health	Very good	104 (22.8)	10 (11.0)	29 (19.5)	65 (30.0)	**25.79 (8)**	**<0.01**
	Good	252 (55.1)	54 (59.3)	83 (55.7)	115 (53.0)		
	Fair	88 (19.3)	24 (26.4)	28 (18.8)	36 (16.6)		
	Bad	11 (2.4)	2 (2.2)	8 (5.4)	1 (0.5)		
	Very bad	2 (0.4%)	1 (1.1%)	1 (0.7%)	0 (0.0%)		
Pregnancy intention	Planned	305 (66.9)	63 (70.0)	95 (63.8)	147 (67.7)	1.594 (4)	0.81
	Unplanned	58 (12.7)	12 (13.3)	20 (13.4)	26 (12.0)		
	Ambivalent	93 (20.4)	15 (16.7)	34 (22.8)	44 (20.3)		
Mode of delivery	Vaginal delivery	237 (51.9)	51 (56.0)	79 (53.0)	107 (49.3)	3.44 (6)	0.75
	Assisted delivery (forceps/ventouse delivery)	59 (12.9)	13 (14.3)	21 (14.1)	25 (11.5)		
	Planned caesarean section	80 (17.5)	14 (15.4)	23 (15.4)	43 (19.8)		
	Emergency caesarean section	81 (17.7)	13 (14.3)	26 (17.4)	42 (19.4)		
Breastfeeding	Any duration of breastfeeding	328 (71.8)	63 (69.2)	113 (75.8)	152 (70.0)	1.83 (2)	0.40
	No breastfeeding	129 (28.2)	28 (30.8)	36 (24.2)	65 (30.0)		
Resumption of sexual activity	Less than 1 week later	1 (0.2)	0 (0.0)	0 (0.0)	1 (0.5)	9.33 (8)	0.32
	1–4 weeks later	52 (11.4)	10 (11.0)	13 (8.7)	29 (13.4)		
	5–8 weeks later	156 (34.1)	25 (27.5)	51 (34.2)	80 (36.9)		
	More than 8 weeks later	238 (52.1)	53 (58.2)	84 (56.4)	101 (46.5)		
	N/A	10 (2.2)	3 (3.3)	1 (0.7)	6 (2.8)		

Significant results are highlighted in bold text.

IMD, Index of Multiple Deprivation; N/A, not available.

### Quantitative analysis

There was no significant association between delivery cohort and most demographic and pregnancy-related characteristics (table 1). Comparing those who delivered in 2020 and 2022, in 2022, there were more respondents in relationships (2020: 26.7%, n=24; 2022: 45.2%, n=98), and fewer married (2020: 61.1%, n=55; 2022: 47.9%, n=104) and single respondents (2020: 8.9%, n=8; 2022: 2.8% n=6 (χ^2^=14.2, p=0.03)). There was a significant increase in respondents self-reporting very good physical health between 2020 (11.0% n=10), 2021 (19.5%, n=29) and 2022 (30.0%, n=65 (χ^2^=25.79, p=0.001)). There was a decrease in respondents reporting good or fair physical health in 2020 (59.3%, n=54 and 26.4%, n=24, respectively), 2021 (55.7%, n=83 and 18.8%, n=28, respectively) and 2022 (53.0%, n=115 and 16.6%, n=36, respectively).

There was no significant association between uptake of any individual or grouped contraceptive method and period of delivery, or between access to preferred PNC type and period of delivery (see table 2). However, there was an observed increase in uptake of the progestogen-only contraceptive pill between 2020 (8.8%, n=8), 2021 (12.8%, n=19) and 2022 (13.4%, n=29) and a decrease in uptake of the combined contraceptive pill between 2020 (11%, n=10), 2021 (4.7%, n=7) and 2022 (5.1%, n=11). In 2020, around a third of respondents reported using no PNC (33.3%, n=30), compared with 23.5% (n=35) in 2021 and 27.6% (n=60) in 2022. However, these differences did not meet the threshold for statistical significance and as such should be interpreted with caution.

**Table 2:** Uptake of postnatal contraception by individual contraceptive type and grouped contraceptive type (healthcare professional prescribed/administered and long acting reversible contraceptive (LARC)) methods

			Year, deliveries occurring between April and June				
**Contraceptive choice**(**within 8 weeks**)		**Total Sample**	**2020n (%**)	**2021n (%**)	**2022n (%**)	**OR**	**95% CI**
Progestogen-only contraceptive pill		56 (12.3)	8 (8.8)	19 (12.8)	29 (13.4)	2020: ref2021: 1.522022: 1.60	2021: 0.63 to 3.622022: 0.70 to 3.65
Combined contraceptive pill		28 (6.1)	10 (11.0)	7 (4.7)	11 (5.1)	2020: ref2021: 0.402022: 0.43	2021: 0.15 to 1.092022: 0.18 to 1.06
Contraceptive pill(type unknown)		23 (5.0)	5 (5.5)	9 (6.0)	9 (4.1)	2020: ref2021: 1.112022: 0.74	2021:0.36 to 3.412022: 0.24 to 2.29
Contraceptive injection		29 (6.3)	4 (4.4)	11 (7.4)	14 (6.5)	2020: ref2021: 1.732022: 1.50	2021: 0.54 to 5.622022: 0.48 to 4.69
Contraceptive implant		25 (5.5)	6 (6.6)	7 (4.7)	12 (5.5)	2020: ref2021: 0.702022: 0.83	2021: 0.23 to 2.152022: 0.30 to 2.28
Emergency contraception (morning after pill)		5 (1.1)	1 (1.1)	1 (0.7)	3 (1.4)	2020: ref2021: 0.612022: 1.26	2021: 0.04 to 9.842022: 0.13 to 12.29
Hormonal coil(intrauterine system, IUS)		10 (2.2)	3 (3.3)	3 (2.0)	4 (1.8)	2020: ref2021: 0.602022: 0.55	2021: 0.12 to 3.052022: 0.12 to 2.51
Copper coil(intrauterine device, IUD)		6 (1.3)	1 (1.1)	1 (0.7)	4 (1.8)	2020: ref2021: 0.612022: 1.69	2021: 0.04 to 9.842022: 0.19 to 15.33
Tubal ligation		6 (1.3)	1 (1.1)	1 (0.7)	4 (1.8)	2020: ref2021: 0.612022: 1.69	2021: 0.04 to 9.842022: 0.19 to 15.33
Vaginal ring		2 (0.4)	1 (1.1)	1 (0.7)	0 (0.0)	2020: ref2021: 0.612022: N/A	2021: 0.04 to 9.842022: N/A
Male condom		139 (30.4)	27 (29.7)	41 (27.5)	71 (32.7)	2020: ref2021: 0.902022: 1.15	2021: 0.51 to 1.602022: 0.68 to 1.96
Male partner vasectomy		7 (1.5)	1 (1.1)	3 (2.0)	3 (1.4)	2020: ref2021: 1.852022: 1.26	2021: 0.19 to 18.052022: 0.13 to 12.29
Fertility awareness apps		7 (1.5)	1 (1.1)	2 (1.3)	4 (1.8)	2020: ref2021: 1.222022: 1.69	2021: 0.11 to 13.702022: 0.19 to 15.33
Withdrawal method		24 (5.3)	1 (1.1)	11 (7.4)	12 (5.5)	2020: ref2021: 7.172022: 5.27	2021: 0.91 to 56.532022: 0.68 to 41.13
Avoiding penetrative sex		15 (3.3)	4 (4.4)	6 (4.0)	5 (2.3)	2020: ref2021: 0.912022: 0.51	2021: 0.25 to 3.332022: 0.14 to 1.96
None		125 (27.4)	30 (33.0)	35 (23.5)	60 (27.6)	2020: ref2021: 0.622022: 0.78	2021: 0.35 to 1.112022: 0.46 to 1.32
Any healthcare professional prescribed contraception		187 (40.9)	39 (42.9)	61 (40.9)	87 (40.1)	2020: ref2021: 0.922022: 0.89	2021: 0.55 to 1.572022: 0.54 to 1.47
Any LARC		70 (15.3)	14 (15.4)	22 (14.8)	34 (15.7)	2020: ref2021: 0.952022: 1.02	2021: 0.46 to 1.972022: 0.52 to 2.01
Preferred contraception method?	Yes	235(53.5)	47 (54.0)	72 (50.3)	116 (55.5)	2020: ref2021: 1.202022: 1.142020: ref2021:1.142022: 0.85	2021: 0.54 to 2.652022: 0.55 to 2.412021: 0.63 to 2.062022: 0.49 to 1.50
	No	68 (15.5)	12 (13.8)	22 (15.4)	34 (16.3)		
	N/A	136 (31.0)	28 (32.2)	49 (34.3)	59 (28.2)		

N/A, not available.

Postnatal healthcare provider contacts are described in table 3. Women who delivered in 2020 had significantly fewer postnatal community midwife contacts (χ^2^=46.04, p<0.001), fewer postnatal health visitor contacts (χ^2^=67.32, p<0.001) and were less likely to have a 6–8 week postnatal check (χ^2^=8.72, p=0.01) than women who delivered in 2022. Women who delivered in 2020 had significantly fewer total community healthcare provider appointments than women who delivered in 2021 and 2022 (χ^2^=71.90, p<0.001).

**Table 3:** Healthcare provider contacts/discussions during the antenatal/postnatal period

			Year, deliveries occurring between April to June				
Postnatal contraceptive contact		**Total**	**2020n (%**)	**2021n (%**)	**2022n (%**)	**X^2^ (d.f.)**	**P value**
Male partner involved in postnatal contraception (PNC) discussions?	Yes	62 (15.9)	5 (6.8)	12 (9.4)	45 (24.1)	**17.99 (2)**	**<0.001**
	No	327 (84.1)	69 (93.2)	116 (90.6)	142 (75.9)		
Male options discussed?	Yes	33 (7.8)	3 (3.7)	11 (8.0)	19 (9.3)	2.55 (2)	0.28
	No	390 (92.2)	78 (96.3)	127 (92.0)	185 (90.7)		
If LSCS, was PNC offered?	Yes	31 (26.7)	4 (18.2)	7 (19.4)	20 (34.5)	3.58 (2)	0.17
	No	85 (73.3)	18 (81.8)	29 (80.6)	38 (65.5)		
Postnatal ward—PNC offered/discussed?	Yes	284 (62.8)	38 (42.7)	94 (63.5)	152 (70.7)	**21.18 (2)**	**<0.001**
	No	168 (37.2)	51 (57.3)	54 (36.5)	63 (29.3)		
Summed no. of home and community midwife visits	0	12 (2.8)	7 (8.2)	3 (2.1)	2 (1.0)	**46.04 (12)**	**<0.001**
	1	42 (9.6)	19 (22.4)	12 (8.4)	11 (5.3)		
	2	94 (21.6)	23 (27.1)	34 (23.8)	37 (17.8)		
	3	105 (24.1)	15 (17.6)	36 (25.2)	54 (26.0)		
	4	66 (15.1)	9 (10.6)	22 (15.4)	35 (16.8)		
	5	42 (9.6)	3 (3.5)	15 (10.5)	24 (11.5)		
	6+	75 (17.2)	9 (10.6)	21 (14.7)	45 (21.6)		
Total no. of health visitor visits	0	19 (4.4)	12 (14.1)	2 (1.5)	5 (2.4)	**67.32 (10)**	**<0.001**
	1	66 (15.4)	28 (32.9)	23 (17.0)	15 (7.2)		
	2	155 (36.1)	23 (27.1)	56 (41.5)	76 (36.4)		
	3	92 (21.4)	7 (8.2)	31 (23.0)	54 (25.8)		
	4	45 (10.5)	8 (9.4)	9 (6.7)	28 (13.4)		
	5+	52 (12.1)	7 (8.2)	14 (10.4)	31 (14.8)		
Total no. of community appointments	1–2	24 (5.8)	18 (22.8)	5 (3.8)	1 (0.5)	**71.90 (4)**	**<0.001**
	3	42 (10.2)	16 (20.3)	15 (11.5)	11 (5.4)		
	4+	347 (84.0)	45 (57.0)	111 (84.7)	191 (94.1)		
Postnatal review with an obstetrician/gynaecologist	Yes	17 (3.7)	3 (3.3)	4 (2.7)	10 (4.6)	0.99 (2)	0.61
	No	439 (96.3)	88 (96.7)	145 (97.3)	206 (95.4)		
Postnatal check at 6–8 weeks	Yes	404 (88.4)	74 (81.3)	129 (86.6)	201 (92.6)	**8.72 (2)**	**0.01**
	No	53 (11.6)	17 (18.7)	20 (13.4)	16 (7.4)		
Specialist SH/contraception service	Yes	19 (4.2)	4 (4.4)	4 (2.7)	11 (5.2)	1.34 (2)	0.51
	No	432 (95.8)	87 (95.6)	144 (97.3)	201 (94.8)		

Significant results are highlighted in bold text.

LSCS, Lower segment Caesarean section; SH, sexual health.

Women who delivered in 2021 and 2022 were significantly more likely to discuss PNC with a healthcare provider and to be offered PNC on the postnatal ward than women who delivered in 2020 (2021: 64.5%, n=94; 2022: 70.7%, n=152 vs 2020: 42.7%, n=38 (χ^2^=21.18, p<0.001)). Women who delivered in 2022 were significantly more likely to report that male partners had been involved in PNC discussions than women who delivered in 2020 and 2021 (24.1%, n=45 vs 6.8%, n=5 and 9.4%, n=12 (χ^2^=17.99, p<0.001)).

### Qualitative analysis

Thematic analysis of the qualitative data identified three overarching themes. Illustrative participant quotations are given in table 4.

**Table 4:** Participant quotations from qualitative data analysis

Theme 1	*A perception that COVID-19 had a negative impact on PNC care*	
	2020	*Access to contraceptives I would of hoped would of been easier had COVID-19 not prevented the lack of appointments and minimal face to face contact with health professionals following giving birth*.
		*Ok but limited. I wished to access the coil but due to COVID-19 my GP was only offering pill or condoms*.
		*I found it difficult due to COVID-19 as ideally I wanted the implant back in but couldn’t get this due to no face-to-face appointments*.
	2021	*It was discussed after the birth of my daughter. However, when I needed contraception my health visitor was not coming anymore due to COVID-19*.
		*Due to COVID-19 there was a lot of reason about who could and couldn’t fit it*.
**Theme 2**	***Inconsistent experiences of PNC care across all three periods***	
**Access**	2020	*I feel that I had no issues accessing contraception after the birth of my child*.
		*Wanted an IUD however waiting list for sexual health service is huge so waited 7 months for this*.
	2021	*I was offered contraception at every appointment and could easily access*.
		*I was well informed about the contraception methods however was unable to access it straight away due to waiting list and had to wait at least 6 month*.
	2022	*I thought it was very easy to get my contraception*.
		*I knew from the start I wanted to have the implant reinserted. Access to this was extremely slow and there was a 6 month waiting list*.
**Information**	2020	*Not discussed by any healthcare professional*.
		*My GP rang me to discuss options. Was really helpful that he called as having a new born I hadn’t had time to contact him myself*.
	2021	*I didn’t get asked or told any information*.
		*Frequently discussed by many professionals who were knowledgeable and helpful*.
	2022	*No information has been provided at any time during my pregnancy or since*.
		*Very satisfied. Enough information was given to help me make a choice*.
**Timing**	2020	*Discussing contraception in the hospital, a few days after having a baby was not an ideal time*.
		*Amazing. Someone came to the ward following my c section and gave me the implant. I hadn’t even thought about contraception following the birth. Took all of the stress and worry away*.
	2021	*It was OK but was only mentioned once by one health care worker who came the day after birth. However, for me it wasn't a priority at the time because I was so wrapped up with being a new mum and tiredness. It would have been good to have a scheduled GP check-up some 8 weeks later where this could have been discussed then when the timing would have been better*.
		*I thought it was really good the injection was offered after the birth even though I didn’t go ahead*.
	2022	*Felt a bit uncomfortable being offered contraception so soon after giving birth in hospital*.
		*Following a c section, it does feel a little rushed and it added more pain to my already bruised body, having said that, it saved me having to worry about arranging this myself weeks later*.
**Theme 3**	***Feeling pressured to accept offers of PNC***	
	2020	*Very easy to access, but felt almost pushy. Like leaving hospital without contraception already in place wasn't an option*.
		*Midwives we’re very forceful on the use of contraception following the day of discharge, it came across as ‘pushy’ and not very informative as to the best contraceptive route to go down. Health visiting team seemed to force the option of contraceptive coil despite my own feelings of not wanting to have this, again was not very informative and told ‘it’s not that bad’ regarding insertion*.
	2021	*I feel I was pressured into getting contraception 12 hours after giving birth*
		*I felt under enormous pressure to get onto contraception after my baby. The GP really wanted me to have the coil fitted, at one point I was thinking -is she on commission!?*
		*Also felt pressured by the GP at my 6–8 week check up to go back onto hormonal contraception*.
	2022	*I felt so much pressure to use contraception even after I explained myself to each individual that I did not want contraception*.
		*Doctor was pushing the pill but I wanted coil*.

1. A perception that COVID-19 had a negative impact on PNC care:
  Some respondents felt that the COVID-19 pandemic had had a negative impact on their ability to access PNC care, especially women in the 2020 cohort. Perceived impacts included increased waiting times for care, reduced contact (especially face-to-face contact) with healthcare professionals (HCPs) and reduced availability of preferred contraceptive options. Only a few responses in 2021 explicitly mentioned COVID-19, and no responses in 2022 explicitly referred to the pandemic.
2. Inconsistent experiences of PNC care across all three periods
  Women in all three cohorts shared diverse experiences of PNC care that did not track clearly to year of delivery, with positive and negative experiences of care in all three groups. Experiences of PNC care were mostly related to three aspects of service provision: access, information and timing. Respondents felt that conversations about PNC should be handled sensitively, and some suggested that the kind of PNC care offered should be adapted depending on individual patients’ circumstances.
3. Feeling pressured to accept offers of PNC
  There were frequent reports of participants feeling pressured to receive PNC care. Sometimes this was in relation to accepting any PNC type, and sometimes in relation to receiving a particular method. These experiences were reported across all three periods, and some women were critical of PNC care that they felt had been inappropriately ‘forceful’.

## Discussion

This study found that women who gave birth during the first COVID-19 lockdown had significantly fewer postnatal midwife and health visitor contacts and were significantly less likely to have 6–8 weeks postnatal check than women who gave birth in non-lockdown periods. PNC care on the postnatal ward prior to discharge was also significantly less likely to take place in the lockdown cohort. Despite this, self-reported experiences of PNC care were similar across all three periods with no significant difference in likelihood of PNC uptake between lockdown and non-lockdown groups.

Demographic and pregnancy-related characteristics of participants were similar across the 3-year groups. Respondents in the 2020 group were significantly more likely to be married than those in 2021/2022. Data from the Office for National Statistics report a 61.0% decrease in marriages across England and Wales in 2020 (n=85 770) compared with 2019 (n=219 850) and fewer marriages in 2021 (n=207 708) than 2019 (n=246 897).^18^ This may in part explain the significant decrease in married respondents in non-lockdown cohorts, as access to marriage was restricted. A significant increase in male partner involvement in PNC discussions within the 2022 group (24.1%) in comparison to the 2020 group (6.8%) and 2021 group (9.4%) may be linked to restrictions on family and partner visits during COVID-19, resulting in male partners may having fewer opportunities to attend appointments in person.^15^ However, given the shift to telemedicine, and the introduction of furlough and work from home schemes, opportunities for male partner involvement in PNC discussions may still have been available in circumstances where they were requested and wanted.

Women who delivered in lockdown were significantly less likely to have various types of postnatal HCP contact, such as midwife/health visitor contact, the 6–8 week GP check and PNC care on the postnatal ward than women who gave birth in 2021/2022. Guidance released by the Royal College of Midwives and the Royal College of Obstetricians and Gynaecologists (RCOG) on 30 March 2020 recommended that women should receive a minimum of three postnatal contacts.^19^ In this study, women who delivered in 2020 were significantly more likely to receive the minimum or fewer than the minimum recommended number of contacts as compared with women who delivered in 2021/2022. In March 2020, one in five midwife roles were unstaffed due to COVID-19, self-isolation, and existing staff vacancies, and 78% of midwifery leads reported pausing face-to-face antenatal and postnatal visits.^20^ Our findings suggest that this had a direct effect on the postnatal care that women received at this time.

Many women in the 2020 group discussed the impact of COVID-19 on their PNC care in the open-ended question responses, while relatively few women in the 2021/2022 groups explicitly mentioned COVID-19. Barriers to PNC care described by participants were perceived to be repercussions of COVID-19 and align with those described elsewhere in the pandemic qualitative literature.^21^ However, evidence presented at the COVID-19 inquiry suggests that NHS services faced significant challenges prepandemic, and that the events of 2020 simply served to place increased pressure on a system that was already under significant strain.^22^ The RCOG’s Better for Women report in 2019 had identified shortcomings in PNC provision and recommended implementing PNC as part of the routine maternity pathway and improving access to the full range of contraception methods.^23^ Prepandemic studies have shown that although a majority (96.7%) of postpartum women reported not planning another pregnancy within the year, only a minority (12.8%) planned to obtain a LARC.^24^ Other studies have reported that only a small proportion (14%) of postpartum women left maternity services with a plan to access to PNC.^25^ Barriers to PNC provision identified in the literature include heavy workloads for staff with short postpartum hospital stays, PNC not being a priority for many women, and systemic issues such as staffing levels and the time taken to insert LARCs. In light of this, the available evidence suggests that challenges in delivering good PNC care were exacerbated, rather than caused, by the pandemic.

COVID-19 could have represented an opportunity to redesign service pathways and to implement PNC care as standard.^26^ Some efforts were made to grasp these opportunities, for example, by making it easier for HCPs to issue the progestogen-only pill, which has fewer contraindications than the combined pill, and by issuing guidance around HCP discussions about PNC. However, the evidence presented here suggests that these changes did not meaningfully improve the PNC experience for women in this sample, in relation to either uptake of PNC or satisfaction with PNC services in 2021/2022.^27^ Indeed, although many women reported conversations with HCPs about PNC, these conversations were frequently described as unwelcome and potentially coercive, and this was consistent across the three participant cohorts.

This is the first UK study to explore experiences of PNC care during the first COVID-19 lockdown. In using qualitative and quantitative methods, it shares women’s experiences that shed light on the quantitative findings. The large geographical reach and demographic diversity of the survey population are also strengths. However, although the PoCo study had a relatively large participant sample, this subanalysis does not. As such, the reduced statistical power associated with a smaller sample may have increased the likelihood of type 2 errors. As per our previous paper, the sample was representative of the background population in NENC ICS in relation to socioeconomic status and age.^14^ However, the lack of diversity of the sample in relation to gender identity and ethnicity is a limitation. As an online survey, there was the potential for digital bias, but this was partially overcome by offering a paper version if required, and there may potentially have been some recall bias when participants were asked to describe events that occurred several years earlier. MM’s male gender and clinical background in obstetrics are acknowledged in the interpretation of the qualitative results, as are the limitations of survey methods for rigorous qualitative exploration. Within this subanalysis, a comparison to a 2019 cohort would have been instructive in better understanding how COVID-19 impacted PNC care in relation to what had come before. In the absence of this or earlier comparison groups, the direct impact of COVID-19 on PNC care cannot be explicitly determined, but rather hypothesised. There remains the possibility that some associations that are reported in this analysis are linked to confounding factors that are not fully explored in this analysis. While the focus of this paper and discussion has been on the impact of COVID-19 on healthcare provider practices, the pandemic also affected patient/individual behaviours that may have resulted in changes to how contraception/postnatal care was accessed. For example, new parents may have been reluctant to host HCPs in their homes and may have experienced barriers to the transition to telemedicine as an alternative. It may also have resulted in changes to sexual behaviours that were not explored in this survey.^28^ Future research might explore these individual-level postnatal experiences of the pandemic using in-depth interviews rather than survey methods.

Our findings suggest that women who delivered in April–June 2020 received significantly less postnatal care than women who delivered in 2021/2022, and these women and their offspring/families may benefit from additional support now as a result. Cohort studies should continue to monitor the outcomes and experiences of children born during the pandemic. Policy-makers should carefully consider how access to postnatal care might be supported and prioritised in future crisis situations, given the potential for significant intergenerational public health harm when early years support is removed. As per the wider PoCo study dataset, this analysis also sheds some light on how PNC care in England might be made more patient-centred: by cutting down on repetitive PNC conversations by asking women if these conversations have already taken place and documenting these conversations appropriately; by making women feel less pressured into receiving PNC and supporting them to access reliable information on the full range of PNC methods and having PNC conversations at the right time; and by having respectful conversations when women choose not to access PNC.

Although the first COVID-19 pandemic lockdown appears to have had a significant impact on new mothers’ access to postnatal care resources, this did not substantively affect access to, or experiences of, PNC care for women in this sample. This most likely reflects shortcomings in the provision of PNC care that preceded the pandemic, rather than successes on the part of healthcare organisations in maintaining services in restricted circumstances. However, these women and families may benefit from additional support postpandemic to mitigate the potential life course implications of restricted support in the postpartum period. While there are improvements that HCPs can make to enhance the experience of PNC care and postnatal care, systematic change to current models of provision and better preparation for future crisis situations are required to ensure that women consistently receive good contraception care. Pandemic opportunities to improve access to PNC care appear to have had modest impact, and future research should continue to evaluate the longer-term impacts of these changes in non-pandemic contexts. Policy-makers and healthcare providers should continue to explore innovative and patient-centred approaches to improving this important element of a postnatal public health offer.

## Supplementary material

10.1136/bmjopen-2024-095608 online supplemental file 1

## Data Availability

Data are available on reasonable request.
