# Supplementary material for: Experiences of postnatal contraceptive care during the COVID-19 pandemic: a multimethods cross-sectional study
Source: BMJ Open. 2025 Jun 17;15(6):e095608. doi: 10.1136/bmjopen-2024-095608 (PMC12182104; doi:10.1136/bmjopen-2024-095608)
Supplement: online supplemental file 1 [file bmjopen-15-6-s001.pdf]

Contraception after you’ve had a baby in the North East and North Cumbria: The PoCo Study

Page 1: Welcome

Thank you for taking the time to complete this survey. It should take around 30 minutes to complete.

This survey will explore your experiences of accessing contraception care in the period after you have had a baby, and the information you provide will help us to understand how and where women in the North East and North Cumbria currently access postnatal contraception. We know that lots of women struggle to access contraception care and family planning advice in the weeks and months after they give birth - understanding where barriers to contraception care exist (as well as where services are doing well) will help us to deliver better services for new mothers across our region. Some of the questions we ask might not be relevant to your experience - where this is the case, please leave these questions blank and move on to the next section. For some questions, you might find it helpful to look at your baby's personal child health record ('red book').

The information you provide will be included in a report that will be shared with healthcare organisations across the North East and North Cumbria, as well as nationally with leadership staff at the Department of Health and Social Care. It will also be included in academic journal articles and conference presentations. Your responses will be anonymous - you will not be identifiable in any of the information we share.

At the end of the survey, you will be asked if you would like to be entered into a prize-draw for the chance to win a £50 shopping voucher.

More information about this research is available to read at **Participant Information Leaflet (survey)**. If you have any questions or comments about this survey, please email **poco.study@newcastle.ac.uk**.

Thank you again for supporting us with this important work - we really appreciate your contribution.

Page 2: Participant consent

1. Please read the following statements and select each of them to indicate that you give your consent to participate in this survey. ✱ *Required*

Please select at least 4 answer(s).

- [ ] I understand that my participation is voluntary and that I am free to withdraw at any time.
- [ ] I understand that data collected during the study will be looked at by authorised individuals from Newcastle University, where it is relevant to my taking part in this research. I give permission for these individuals to have access to these data.
- [ ] I consent for the data I provide to be described and discussed in published reports and academic journal articles, and understand that I will not be identifiable from this information.
- [ ] I consent to take part in this study.

Page 3: Screening questions

2. Are you aged 16 or over?

- Yes
- No

3. Have a you had a baby in the last three years?

- Yes
- No

4. Was your baby born in the North East or North Cumbria?

- Yes
- No

If you have answered **YES** to all three of these questions, you can complete this survey - please continue to the next page.

If you have answered **NO** to any of these questions, unfortunately you are not able to take part in this survey. Thank you for the interest you have shown in the project - if you would still like to share or discuss your experiences of any of the care that you received during your pregnancy, please contact your hospital PALS team, or email the research team at **poco.study@newcastle.ac.uk**.

5. Were you asked to take part in this survey by a healthcare professional? If so, in what role and/or in what setting does the healthcare professional work?

- No (not applicable)

- Yes - by a health visitor
- Yes - by a community midwife
- Yes - by a GP/practice nurse (please specify the GP practice in the 'other' box below)
- Yes - by staff at the Royal Victoria Infirmary (RVI), Newcastle
- Yes - by staff at Sunderland Royal Infirmary (SRH), Sunderland
- Yes - by staff at University Hospital of North Durham (UHND), Durham
- Yes - by staff at Darlington Memorial Hospital (DMH), Darlington
- Yes - by staff at The Queen Elizabeth (QE) Hospital, Gateshead
- Yes - by staff at Northumbria Specialist Emergency Care Hospital (NSECH), Cramlington
- Yes - by staff at North Tees Hospital (NTH), Stockton
- Yes - by staff at University Hospital of Hartlepool (UHH), Hartlepool
- Yes - by staff at James Cook University Hospital, Middlesbrough
- Yes - by staff at South Tyneside District Hospital (STDH), South Shields
- Yes - by staff at Cumberland Infirmary, Carlisle
- Yes - by staff at West Cumberland Hospital, Whitehaven
- Other

5.a. If you selected 'Other' or 'Yes - by a GP/practice nurse', please specify:

Page 4: About you

This section asks questions about you - about your background, about where you live, and about your education and work (if applicable). This information helps us to understand whether or not people from particular social groups are more or less likely to have good experiences of postnatal contraception care.

6. How old are you?

- 16
- 17
- 18
- 19
- 20
- 21
- 22
- 23
- 24
- 25
- 26
- 27
- 28
- 29
- 30
- 31
- 32
- 33
- 34
- 35
- 36
- 37
- 38
- 39

- 40
- 41
- 42
- 43
- 44
- 45
- 46
- 47
- 48
- 49
- 50
- 51+
- Prefer not to say

7. How would you describe your gender?

- Female
- Trans man
- Non-binary
- Other
- Prefer not to say

7.a. If you selected Other, please specify:

8. How would you describe your sexual orientation?

- Straight (heterosexual)
- Gay/lesbian (homosexual)

- Bisexual
- Other
- Prefer not to say

8.a. If you selected Other, please specify:

9. How would you describe your current relationship status?

- Married
- In a civil partnership
- In a relationship
- Single
- Other
- Prefer not to say

9.a. If you selected Other, please specify:

10. What is your ethnicity?

- White English/Welsh/ Scottish/Northern Irish/British
- Irish
- Gypsy or Irish Traveller
- Any other white background
- White and Black Caribbean
- White and Black African
- White and Asian

- Any other mixed/multiple ethnic background
- Indian
- Pakistani
- Bangladeshi
- Chinese
- Any other Asian background
- Black African
- Black Caribbean
- Any other Black/African/Caribbean background
- Arab
- Other
- Prefer not to say

10.a. If you selected Other, please specify:

11. In which country were you born?

12. If you were not born in the UK, how old were you when you moved here? *(If this question is not applicable to you, please leave blank and move on to question 13)*

13. What is your postcode?

14. Which of the following best describes your highest educational qualification:

- ◯ 5 or more GCSEs (A*-C, 9-4), O levels (passes) or CSEs (grade 1)
- ◯ Any other GCSEs, O levels or CSEs (any grades) or Basic Skills course
- ◯ 2 or more A levels, 4 or more AS levels
- ◯ 1 A level, 2-3 AS levels
- ◯ 1 AS level
- ◯ NVQ level 3, BTEC National, OND or ONC, City and Guilds Advanced Craft
- ◯ NVQ level 2, BTEC General, City and Guilds Craft
- ◯ NVQ level 1
- ◯ No qualifications
- ◯ Undergraduate degree
- ◯ Higher degree (MA, MSc, PhD)
- ◯ Other

14.a. If you selected Other, please specify:

15. Thinking back to when you became pregnant most recently, which of the following best describes your employment status at that time (you can choose multiple options):

- ☐ Going to school or college full-time (including on vacation)
- ☐ In paid employment (or temporarily away)- not self-employed
- ☐ In paid employment (or temporarily away)- self-employed
- ☐ On a Government scheme for employment training
- ☐ Doing unpaid work for a business that you own, or that a relative owns
- ☐ Waiting to take up paid work already obtained

- [ ] Looking for paid work or a Government training scheme
- [ ] Intending to look for work but prevented by temprorary sickness or injury (if more than 28 days, select 'permanently unable to work' below)
- [ ] Permanently unable to work because of long-term sickness or disability
- [ ] Retired from paid work
- [ ] Looking after home or family
- [ ] Other

15.a. If you selected Other, please specify:

____

16. What is your current total household income (before tax)?

- ○ Less than £10,000 per year
- ○ £10,000-£19,999 per year
- ○ £20,000-£29,999 per year
- ○ £30,000-£39,999 per year
- ○ £40,000-£49,999 per year
- ○ £50,000-£69,999 per year
- ○ £70,000-£100,000 per year
- ○ More than £100,000 per year
- ○ Prefer not to say

17. How would you describe your physical health?

- ○ Very good
- ○ Good
- ○ Fair
- ○ Bad

- Very bad

18. How would you describe your mental health and wellbeing? *(If you are struggling with your mental health and require support, a list of support organisations is provided at the end of this survey)*

- Very good
- Good
- Fair
- Bad
- Very bad

19. Do you have a disability?

- Yes
- No
- Prefer not to say

19.a. If you answered yes, can you describe this?

20. Do you smoke tobacco?

- No - never smoked
- No - used to smoke, but quit
- Yes - current smoker

Page 5: Your reproductive health history

This section asks questions about all of your previous pregnancies, and about how you have accessed contraception in the past.

Not all pregnancies have a positive outcome, and pregnancy and childbirth can be difficult and distressing experiences for many women. We have included a list of support organisations at the end of this survey that are there to help if you are struggling with any of the topics discussed in the questions that follow.

21. How many times have you been pregnant? Please record how many times you have experienced the following pregnancy outcomes *(please enter a number for all that apply)*:

	How many times have you experienced this pregnancy outcome?
Livebirth	
Stillbirth	
Miscarriage at less than 12 weeks gestation	
Miscarriage at more than 12 weeks gestation	
Ectopic pregnancy (when a fertilised egg implants itself outside of the womb, usually in one of the fallopian tubes)	
Molar pregnancy (when there's a problem with a fertilised egg, which means a baby and a placenta do not develop the way they should after conception)	
Pregnancy ended in abortion (termination)	

22. How old were you when you became pregnant for the first time?

23. Which of the following methods of contraception have you used at any time in the past *(please select all that apply)*?

- ☐ Never used any form of contraception
- ☐ Male condom
- ☐ Female condom
- ☐ Cap/diaphragm
- ☐ Partner has been sterilised (had a vasectomy)
- ☐ I have been sterilised (tubal ligation, 'tubes tied')
- ☐ Combined oral contraceptive pill
- ☐ Progesterone only contraceptive pill
- ☐ Contraceptive pill - don't know which type
- ☐ Hormonal coil (e.g. Mirena, Jaydess, Kyleena, Levosert)
- ☐ Copper coil/intra-uterine device (IUD)
- ☐ Vaginal ring (e.g. NuvaRing)
- ☐ Contraceptive patch (e.g. EVRA)
- ☐ Injections
- ☐ Implant
- ☐ Emergency contraceptive pill/morning after pill
- ☐ Emergency copper coil/intra-uterine device (IUD)
- ☐ Safe period/ calendar method/ rhythm method
- ☐ Withdrawal (not ejaculating in a partner's vagina)
- ☐ Fertility awareness apps or devices
- ☐ Avoiding penetrative sex
- ☐ Spermicides (foams/gels/sprays/pessaries)
- ☐ Lactational amenorrhoea method (LAM - breastfeeding as 'natural' contraception))
- ☐ Other

23.a. If you selected Other, please specify:

24. Where have you accessed contraception from in the past (please select all that apply)?

- ☐ GP practice
- ☐ Sexual health clinic
- ☐ Contraception clinic
- ☐ Hospital
- ☐ Other

24.a. If you selected Other, please specify:

25. Where would you prefer to go to access contraception (please select all that apply)?

- ☐ GP practice
- ☐ Sexual health clinic
- ☐ Contraception clinic
- ☐ Hospital
- ☐ Other

25.a. If you selected Other, please specify:

26. Would you say that your contraceptive needs had been met in the past?

- Yes - completely met
- Yes - partially met
- No
- Don't know

Page 6: Your most recent pregnancy

This section asks questions about your most recent pregnancy - the questions that follow, about your postnatal contraception experiences, are in relation to this most recent pregnancy.

27. How old were you when you completed your most recent pregnancy?

28. When did you complete your most recent pregnancy (please select the month AND year)?

- [ ] 2019
- [ ] 2020
- [ ] 2021
- [ ] 2022
- [ ] January
- [ ] February
- [ ] March
- [ ] April
- [ ] May
- [ ] June
- [ ] July
- [ ] August
- [ ] September
- [ ] October
- [ ] November
- [ ] December

29. Did your most recent pregnancy result in a livebirth?

- Yes
- No

30. How many babies did you have in your most recent pregnancy?

- One (singleton pregnancy)
- Two (twins)
- Three (triplets)
- Four (quadruplets)
- Other

30.a. If you selected Other, please specify:

31. Was the pregnancy planned, unplanned or ambivalent (not planned as such but also not taking steps to avoid pregnancy)?

- Planned
- Unplanned
- Ambivalent

32. Were you using contraception at the time of becoming pregnant?

- Yes
- No

32.a. If you answered yes, what type of contraception were you using?

33. Who was primarily responsible for your antenatal care during your pregnancy?

- Midwife (midwife-led care - 'low risk')
- Hospital doctor (obstetrician/consultant-led care - 'high risk')

34. Where did you have your baby/babies?

- At home
- The Royal Victoria Infirmary (RVI), Newcastle
- Sunderland Royal Infirmary (SRH), Sunderland
- University Hospital of North Durham (UHND), Durham
- Darlington Memorial Hospital (DMH), Darlington
- The Queen Elizabeth (QE) Hospital, Gateshead
- Northumbria Specialist Emergency Care Hospital (NSECH), Cramlington
- North Tees Hospital (NTH), Stockton
- University Hospital of Hartlepool (UHH), Hartlepool
- South Tyneside District Hospital (STDH), South Shields
- Cumberland Infirmary, Carlisle
- West Cumberland Hospital, Whitehaven
- Other

34.a. If you selected Other, please specify:

35. How did you give birth to your baby/babies?

- Straightforward vaginal delivery
- Assisted birth (forceps)
- Assisted birth (Ventouse suction cap)
- Planned C-section
- Emergency C-section
- Other

35.a. If you selected Other, please specify:

36. Did you breastfeed your baby/babies? If yes, for how long?

- No
- Yes - for less than six weeks in total
- Yes - for more than six weeks/less than three months in total
- Yes - for between three and six months in total
- Yes - for more than six months in total
- Yes - currently breastfeeding and completed pregnancy less than six weeks ago
- Yes - currently breastfeeding and completed pregnancy more than six week/less than three months ago
- Yes - currently breastfeeding and completed pregnancy between three and six months ago

37. Did you experience any of the following antenatal complications during your pregnancy (please select all that apply)?

- Gestational diabetes
- Pre-eclampsia

- ☐ Pregnancy-induced hypertension (high blood pressure)
- ☐ Obstetric cholestasis (pregnancy-induced liver disease)
- ☐ Placenta praevia
- ☐ Placenta accreta
- ☐ Reduced fetal movements
- ☐ Antepartum haemorrhage (heavy bleeding during pregnancy)
- ☐ IUGR (intrauterine growth restriction)
- ☐ Baby's weight estimated to be below the 10th percentile
- ☐ Baby's weight estimated to be above the 90th percentile
- ☐ PPROM (preterm prelabour rupture of membranes)
- ☐ Other

37.a. If you selected Other, please specify:

____

38. Did you experience any of the following complications during the birth of your baby/babies (please select all that apply)?

- ☐ Placental abruption (where the placenta separates from the wall of the womb)
- ☐ Shoulder dystocia
- ☐ Third- or fourth-degree perineal tear
- ☐ Retained placenta requiring manual removal in theatre
- ☐ Intrapartum haemorrhage (heavy bleeding during delivery)
- ☐ Preterm labour (before 32 weeks gestation)
- ☐ Preterm labour (between 32-37 weeks gestation)
- ☐ Other

38.a. If you selected Other, please specify:

____

39. Did you experience any of the following complications after the birth of your baby/babies (please select all that apply)?

- ☐ Postpartum haemorrhage (heavy bleeding after delivery)
- ☐ Retained placenta
- ☐ Endometritis (infection of the lining of the womb)
- ☐ Perineal infection (following perineal repair for tear/episiotomy, for example)
- ☐ C-section scar infection and/or breakdown
- ☐ Mastitis (breast inflammation/infection)
- ☐ Postnatal depression
- ☐ Other

39.a. If you selected Other, please specify:

____

40. How soon after completing your most recent pregnancy did you resume sexual activity (if applicable)?

- ○ Less than one week later
- ○ 1-4 weeks later
- ○ 5-8 weeks later
- ○ More than 8 weeks later
- ○ Not applicable

41. Did you use any of the following contraception-types within eight weeks of completing your most recent pregnancy? Select all that apply:

- ☐ No (none apply)
- ☐ Male condom
- ☐ Female condom

- Cap/diaphragm
- Partner had been sterilised (had a vasectomy)
- I was been sterilised (tubal ligation, 'tubes tied')
- Combined oral contraceptive pill
- Progesterone only contraceptive pill
- Contraceptive pill - don't know which type
- Hormonal coil (e.g. Mirena, Jaydess, Kyleena, Levosert)
- Copper coil/intra-uterine device (IUD)
- Vaginal ring (e.g. NuvaRing)
- Contraceptive patch (e.g. EVRA)
- Injection
- Implant
- Emergency contraceptive pill/morning after pill
- Emergency copper coil/intra-uterine device (IUD)
- Safe period/calendar method/rhythm method
- Withdrawal (not ejaculating in a partner's vagina)
- Fertility awareness apps or devices
- Avoiding penetrative sex
- Spermicides (foams/gels/sprays/pessaries)
- Lactational amenorrhoea method (LAM - breastfeeding as 'natural' contraception))
- Other

41.a. If you selected Other, please specify:

41.b. If you were given a prescription for contraception or had a coil/implant/injection sited during this period, where did you access this?

- Hospital (during C-section)
- Hospital (postnatal ward)
- Hospital (other)
- GP practice

- [ ] Sexual health clinic
- [ ] Contraception clinic
- [ ] Other

41.b.i. If you selected Other, please specify:

____

41.c. Do you continue to use this contraception-method now?

- ( ) Yes
- ( ) No

41.d. Were you able to access your preferred contraception-method during the eight weeks after completing your most recent pregnancy?

- ( ) Yes
- ( ) No
- ( ) Not applicable - did not want contraception/no preferred method

41.e. If you answered no, why not?

____

Page 7: During your pregnancy

This section asks questions about the care you received during your most recent pregnancy, before you had your baby/babies.

42. During your most recent pregnancy, did any of the following healthcare professionals discuss postnatal contraception with you (please select all that apply)?

- ☐ No
- ☐ Yes - community midwife
- ☐ Yes - hospital midwife
- ☐ Yes - health visitor
- ☐ Yes - obstetrician (hospital doctor)
- ☐ Yes - GP
- ☐ Yes - sexual health doctor/nurse
- ☐ Other

42.a. If you selected Other, please specify:

42.b. If you answered yes, when and where did these conversations take place, and how many weeks pregnant were you at the time?

42.c. Did you make a decision about postnatal contraception at this point?

- ○ No
- ○ Yes - and I was able to access this contraception-method after completing my pregnancy
- ○ Yes - but I wasn't able to access this contraception-method after completing my pregnancy
- ○ Yes - but I later changed my mind

43. If you had a male partner, were male contraception-methods (for example, vasectomy) ever discussed during conversations with healthcare professionals during your pregnancy?

- Yes
- No
- Not applicable

43.a. If you had a male partner, was he ever involved in any discussions you had with healthcare professionals about contraceptive-methods?

- Yes
- No
- Can't remember
- Not applicable

44. If you had a planned C-section, was contraception discussed with you during your pre-op assessment, or when you gave consent for the operation?

- Yes
- No
- Not applicable

44.a. If you answered yes, can you remember what contraception-methods were offered?

Page 8: On the postnatal ward

This section asks questions about the care you received straight after you had your baby/babies while you were still in hospital (if applicable).

45. Before you were discharged home from the postnatal ward, did a healthcare professional speak to you about contraception options?

- ○ Yes
- ○ No
- ○ Not applicable (had home birth)

45.a. If you answered yes, which healthcare professionals had these conversations with you?

- ☐ Midwife
- ☐ Doctor
- ☐ Healthcare assistant
- ☐ Other

45.a.i. If you selected Other, please specify:

____

45.b. Were you satisfied with the information you were given about postnatal contraception before you were discharged home?

- ○ Yes
- ○ No
- ○ Not applicable - no information provided

46. Were you offered access to contraception before you were discharged home?

- Yes
- No

46.a. If you answered yes, which contraception-methods were offered?

46.b. Were you offered your preferred contraception-method?

- Yes
- No
- Not applicable - no contraception offered
- Not applicable - did not want contraception at that point

Page 9: Contact with your community midwife

This section asks questions about contact you had with your community midwife, after you left hospital and returned home.

47. After you had your most recent baby/babies, how many times did a midwife visit you at your home?

48. After you had your most recent baby/babies, how many times did you see a midwife at a location outside of your home (for example, at a post-birth clinic)?

49. At any point after completing your most recent pregnancy did you see a midwife that you knew already/who you had seen during pregnancy?

- Yes
- No

50. After you had your baby and after leaving hospital, did a midwife speak to you about contraception?

- Yes
- No

50.a. If you answered yes, was the midwife who spoke to you about contraception known to you (had you seen her previously)?

- Yes
- No

50.b. Would you prefer to have conversations about postnatal contraception with someone you have seen earlier in your pregnancy?

- Yes
- No
- No preference/don't mind

Page 10: Contact with health visitors

This section asks questions about contact you had with health visitors, during your pregnancy and after you left hospital and returned home.

51. Did you see a health visitor during your most recent pregnancy, before you had your baby/babies?

- Yes
- No
- Can't remember

52. After you had your most recent baby/babies, when did you first see a health visitor?

- Less than a week later
- one to two weeks later
- two to four weeks later
- More than four weeks later

53. In the first three months after you had your baby/babies, how many times did you see a health visitor?

____

54. Did you see the same health visitor each time?

- Yes
- No - I saw 2 health visitors

- No - I saw 3 or more health visitors

54.a. If you answered no, would you have preferred to have seen the same health visitor on each visit?

- Yes
- No
- No preference

55. Did a health visitor discuss postnatal contraception with you?

- Yes
- No

55.a. If you answered yes, did the advice the health visitor offered you help you with your contraceptive choices?

- Yes
- No
- Not applicable

55.b. If you answered yes, did a health visitor support you to access contraception?

- Yes
- No
- Not applicable - already had contraception
- Not applicable - did not want to access contraception at that time

Page 11: Contact with your GP practice

This section asks questions about contact you had with your GP or GP practice nurse 6-8 weeks after you had your baby/babies.

56. Did you have a postnatal check/review appointment at your GP practice 6-8 weeks after completing your most recent pregnancy?

- Yes
- No

56.a. If you answered no, were you advised by a health professional or aware that you should have a 6-8 week postnatal check following the birth of your most recent baby/babies?

- Yes
- No
- Can't remember

56.b. If you answered yes, did you have to make this appointment yourself or did your GP practice automatically make this follow-up appointment for you?

- I organised the postnatal check appointment myself
- The practice automatically gave me an appointment for a postnatal check

56.c. If you answered yes, was this appointment with a GP or a practice nurse?

- GP (doctor)
- Practice nurse

56.d. If you answered yes, was postnatal contraception discussed and offered at this appointment?

- Yes - discussed only

- Yes - discussed and offered
- No

56.d.i. Were you able to access your preferred contraception-method at this appointment?

- Yes - prescription given
- Yes - contraceptive injection given
- Yes - appointment for insertion of coil/implant arranged
- No
- Not applicable - already had contraception
- Not applicable - did not want to access contraception at that time

56.e. If you didn't have a postnatal check with a GP/practice nurse, why weren't you able to access this?

57. If you did access contraception at your GP/practice nurse postnatal check, which contraception-method(s) were you given?

- [ ] Male condom
- [ ] Female condom
- [ ] Cap/diaphragm
- [ ] Referral for vasectomy (for male partner)
- [ ] Referral for sterilisation (tubal ligation, 'tubes tied')
- [ ] Combined oral contraceptive pill
- [ ] Progesterone only contraceptive pill
- [ ] Contraceptive pill - don't know which type
- [ ] Hormonal coil (e.g. Mirena, Jaydess, Kyleena, Levosert)
- [ ] Copper coil/intra-uterine device (IUD)
- [ ] Vaginal ring (e.g. NuvaRing)
- [ ] Contraceptive patch (e.g. EVRA)

- [ ] Injection
- [ ] Implant
- [ ] Spermicides (foams/gels/sprays/pessaries)
- [ ] Other

57.a. If you selected Other, please specify:

Page 12: Emergency/unplanned care

This section asks questions about any unplanned/urgent medical care you received during the 6-8 week period after you completed your most recent pregnancy.

58. Did you access any unplanned/emergency healthcare during the eight weeks after you completed your pregnancy (at A&E, urgent care, your GP practice or your local pregnancy assessment unit, for example)?

- Yes
- No

58.a. If you answered yes, where did you access this care and for what reason(s)?

58.b. If you answered yes, was contraception discussed with you during any of these visits?

- Yes
- No
- Can't remember

58.c. If contraception was discussed, were you able to access contraception during the visit? What contraception method(s) were you offered?

Page 13: Postnatal medical review with a gynaecologist/obstetrician

This section asks questions about any follow-up appointments you had with an obstetrician or gynaecologist after completing your most recent pregnancy.

59. Did you have an appointment to see a gynaecologist or obstetrician at hospital for review during the eight weeks after you completed your pregnancy?

- Yes
- No

59.a. If yes, why was this appointment arranged?

59.b. If yes, was postnatal contraception discussed with you at this appointment?

- Yes
- No

59.c. If contraception was discussed, were you able to access contraception during the visit? What contraception method(s) were you offered?

Page 14: Contact with specialist sexual health/contraception services

This section asks questions about any contact you had with specialist sexual health or contraception services after completing your most recent pregnancy.

60. Did you attend a specialist contraception clinic or service during the eight weeks following the completion of your pregnancy?

- Yes
- No

60.a. If you answered yes, were you able to access the contraception method of your choice through this service?

- Yes
- No

60.b. If you answered yes, which contraception-method did you access at this appointment?

60.c. If you answered yes, when did you arrange this appointment?

- During pregnancy (while pregnant)
- After having baby/babies

60.d. Had you used this service before?

- Yes
- No

Page 15: Your views and experiences

This section gives you the chance to share your views on how contraception should be provided during the postnatal period. Please write as much or as little as you would like to.

61. How would you describe your experiences of accessing contraception and contraception information following your most recent pregnancy?

62. What did services/healthcare professionals do well, and what could they do differently or better to make it easier to access contraception after you've had a baby/babies?

63. With which healthcare professionals would you prefer to have conversations about postnatal contraception? Why would you prefer to have postnatal contraception conversations with these professionals?

64. When during your pregnancy or postnatal period would be the best time to have a discussion about postnatal contraception?

65. Undertaking research with women who have recently had a baby can be challenging for a number of reasons, but we know that the weeks and months after completing a pregnancy are a really important time for mothers and children, and more postnatal research is needed. Can you suggest any ways in which participating in research in the postnatal period (for example, completing surveys such as this, or being interviewed or attending a focus group session) could be made easier, more appealing, and more accessible for new mothers? What support would you like/need to be able to support postnatal research activities? Which areas of postnatal health and care do you think researchers should look at more closely?

Page 16: Thank you

If you would like to be entered into a free prize-draw, for the chance to win a £50 gift voucher, please enter your email address in the box below.

Your email address will not be used for any other purpose, will not be shared with anyone else, and will be deleted by the research team after the prize-draw is made.

66. Enter your email address here:

67. Is there anything else you'd like to say about your experiences of accessing contraception during the postnatal period that hasn't been covered in the survey? Please also use this section to provide feedback on the survey if you would like to.

Page 17: Final page

Thank you for completing this survey - the information that you have shared will help us to develop and deliver postnatal services in our region that are better-equipped to meet the needs of women who have recently had a baby.

If you have any comments or questions, please don't hesitate to contact the principal investigator Dr Malcolm Moffat at **malcolm.moffat@newcastle.ac.uk**.

Support organisations

Contraception/Sexual Health

- https://www.nhs.uk/conditions/contraception/
- https://pregnancyandbirthchoices.co.uk/planning-your-pregnancy/pathway-postnatal/
- https://www.northernlms.org/
- Find sexual health services (www.nhs.uk)

Stillbirth/neonatal death

- Sands | Stillbirth and neonatal death charity

Infant Feeding

- https://www.nhs.uk/start4life/baby/feeding-your-baby/breastfeeding/
- https://www.nhs.uk/conditions/baby/breastfeeding-and-bottle-feeding/bottle-feeding/advice/
- National Breastfeeding Helpline - 0300 100 0212 (available every day, 9:30am to 9:30pm). www.nationalbreastfeedinghelpline.org.uk

Tobacco Dependency

- Free 'Smoke free' app available http://getmesmokefree.com/

Healthy Weight

- Eatwell Guide. https://www.nhs.uk/live-well/eat-well/the-eatwell-guide/
- Active Pregnancy Foundation. www.activepregnancyfoundation.org and Find Your Active | The

APF (activepregnancyfoundation.org)

- NHS Exercise in Pregnancy. Exercise in pregnancy - NHS (www.nhs.uk)
- NHS Keeping fit and healthy with a baby. Keeping fit and healthy with a baby - NHS (www.nhs.uk)
- This Girl Can: Exercising with your baby. Exercising with your baby - This Girl Can
- This Mum Moves (TMM) - Baby Buddy. This Mum Moves | Baby Buddy (babybuddyapp.co.uk)
- This Mum Moves: How can you be more active after childbirth? Post Pregnancy Leaflet3 (thismummoves.co.uk)

Mental Health and Wellbeing

- MIND Mental Health & Pregnancy Resources Mental Health & pregnancy resources from MIND
- Perinatal Depression Film Perinatal Positivity animation Film about Perinatal depression
- MIND Medication in Pregnancy Medication in pregnancy information from Mind
- PND Awareness & Support Pandas PND support and awareness organisation
- MH Information for Families Online MH information for families from Royal College of Psychiatry
- Resources for Dads: Dad's Matter The Book of Man The Dad Pad The Dad Pad Neonatal
- Perinatal Mental Health Specialists | PND counselling | House of Light
- www.digidad.uk
- https://www.neydl.uk/
- Signpost NENC | A hub for North East services
