# Supplementary material for: Experiences of postnatal contraceptive care during the COVID-19 pandemic: a multimethods cross-sectional study
Source: BMJ Open. 2025 Jun 17;15(6):e095608. doi: 10.1136/bmjopen-2024-095608 (PMC12182104; doi:10.1136/bmjopen-2024-095608)
Supplement: Supplementary file 2 [file bmjopen-2024-095608.reviewer_comments.pdf]

PEER REVIEW HISTORY

BMJ Open publishes all reviews undertaken for accepted manuscripts. Reviewers are asked to complete a checklist review form and are provided with free text boxes to elaborate on their assessment. These free text comments are reproduced below.

ARTICLE DETAILS

Title (Provisional)

Experiences of postnatal contraceptive care during the COVID-19 pandemic: A multi-methods cross-sectional study

Authors

Kelly, Shauna; Moffat, Malcolm; Thompson, Caitlin; Jackowich, Robyn; Möller-Christensen, Christine; Sullivan, Claire; Rankin, Judith

---

VERSION 1 - REVIEW

**Reviewer**	**1**
**Name**	**Foley, Kimberley**
**Affiliation**	**Imperial College London, Primary Care and Public Health**
**Date**	**18-Dec-2024**
**COI**	**None**

This study reports on a sub-analysis of a larger survey, specifically reporting on experiences of obtaining postnatal contraception across different pandemic periods. In some areas there was a lack of detail which made it challenging to interpret the results of this work. It would be useful to pull in more literature related to the pre-covid period (if available) to better support the discussion/conclusions in relation to mention of shortcomings of PNC provision. Specific suggestions are provided below:

Abstract:

For clarity, could you please define 'PNC' at first use for the reader? It would also be helpful to include the specific aim for this study in the abstract.

The conclusions in the abstract (and in the main paper) did not seem to be strongly linked to the results of this study as there was no data on pre-covid PNC. Could the conclusions be reworded to better reflect the main findings (in acknowledgement of the data limitations)?

Strengths and limitations:

The authors refer to recall bias, but I wondered if this was recall bias or error? (Is there likely to be differential recall between particular groups that might affect the results and if so, how might this arise?)

Linked to the comment above, the limitations referred to the lack of a pre-covid cohort, but providing a stronger discussion linked to research from pre-covid may help support the statement that there are pre-existing shortcomings in PNC provision.

How this study might affect research, practice or policy:

- Is there any recommendation/suggestion about how this information could be helpful in planning for future pandemics? Or how future postnatal care provision could be tailored to better support birthing parents? (This may mean identifying some additional research recommendations instead of practice/policy recommendations).

Introduction:

- The introduction was quite brief, is it possible to expand on how the covid-19 pandemic affected PNC (if known), and if not known, how the authors feel PNC could have been affected? (E.g. change in provision of services, focus on essential services only, etc?)

- It would also be helpful to have a bit more detail of the PoCo study for additional context.

Methods:

- Details about the methods/survey design were provided in a reference, but it would be helpful to include a short summary here (e.g. the sampling strategy, the structure of the survey, etc. and perhaps the survey could be provided in an appendix?) Was there previous work done to pilot test the survey and/or examine any of the properties (e.g. item discrimination)?

- It's great to see the patient and public involvement in this work.

- I didn't see a statement about ethical approval, is this available and could this be added?

Results:

- Quantitative results were clearly explained. When describing the results of Table 2, selected results were mentioned, but these were based on small numbers and are not statistically significant, which should be mentioned.

- Could you please clarify how much missing data there was and how this was handled?

Discussion:

- As mentioned earlier, additional information about PNC experiences pre-pandemic would be useful (I understand this data was not collected in this study, but experiences from the literature).

- It would be helpful to add a section in describing the potential for confounding in this study and either the implications of this, or how future work could address potential confounding.

- How does this study sample relate to the population in the wider study? Is it possible to discuss the generalizability of this study to the wider population in the ICS?

- These findings are consistent with papers published describing experiences of accessing contraceptive care during lockdowns, and some of this literature could be included/referenced.

- It might be useful to refer the reader to the timeline of lockdowns and a reminder of the social restrictions in place. For example, could the social restrictions during the lockdown period have been the reason why male partners were less involved in PNC discussions? (This broader context may be useful to help interpretation).

Many thanks for the opportunity to review this paper and I hope these comments are useful to the authors.

**Reviewer**	**2**
**Name**	**Ramakrishnan, Jayalakshmy**
**Affiliation**	**JIPMER**
**Date**	**04-Apr-2025**
**COI**	**None**

The study objective was very pertinent, and it was highlighted in the manuscript. However, the data collection through digital medium was a limitation in this study. it was mentioned as well in the manuscript. The qualitative could have been more elaborate, it was not addressing several health system limitations in the study. Another stakeholder meeting could have to added to address the health system's perspective of the problem.

VERSION 1 - AUTHOR RESPONSE

Reviewer: 1
Dr. Kimberley Foley, Imperial College London
Comments to the Author:
This study reports on a sub-analysis of a larger survey, specifically reporting on experiences of obtaining postnatal contraception across different pandemic periods. In some areas there was a lack of detail which made it challenging to interpret the results of this work. It would be useful to pull in more literature related to the pre-covid period (if available) to better support the discussion/conclusions in relation to mention of shortcomings of PNC provision. Specific suggestions are provided below:

Abstract:
For clarity, could you please define 'PNC' at first use for the reader? It would also be helpful to include the specific aim for this study in the abstract.
The conclusions in the abstract (and in the main paper) did not seem to be strongly linked to the results of this study as there was no data on pre-covid PNC. Could the conclusions be reworded to better reflect the main findings (in acknowledgement of the data limitations)?

We have made the changes as suggested.

Strengths and limitations:

The authors refer to recall bias, but I wondered if this was recall bias or error? (Is there likely to be differential recall between particular groups that might affect the results and if so, how might this arise?)

Thank you. We have clarified this point – we think that there is the potential for recall bias and recall error.

Linked to the comment above, the limitations referred to the lack of a pre-covid cohort, but providing a stronger discussion linked to research from pre-covid may help support the statement that there are pre-existing shortcomings in PNC provision.

Thank you. We have expanded our consideration of the pre-COVID literature in the discussion section.

How this study might affect research, practice or policy:
- Is there any recommendation/suggestion about how this information could be helpful in planning for future pandemics? Or how future postnatal care provision could be tailored to better support birthing parents? (This may mean identifying some additional research recommendations instead of practice/policy recommendations).

As per BMJ Open author guidelines, this section has been removed. However, we have expanded on the recommendations shared in the discussion section.

Introduction:
- The introduction was quite brief, is it possible to expand on how the covid-19 pandemic affected PNC (if known), and if not known, how the authors feel PNC could have been affected? (E.g. change in provision of services, focus on essential services only, etc?)

Thank you. We have added some additional detail in relation to this comment.
- It would also be helpful to have a bit more detail of the PoCo study for additional context.

Thank you. We have added some additional information about the PoCo study.

Methods:
- Details about the methods/survey design were provided in a reference, but it would be helpful to include a short summary here (e.g. the sampling strategy, the structure of the survey, etc. and perhaps the survey could be provided in an appendix?) Was there previous work done to pilot test the survey and/or examine any of the properties (e.g. item discrimination)?

We have clarified the points raised and will add the survey template PDF as an appendix/supplementary material.
- It's great to see the patient and public involvement in this work.

Thank you – the group was very supportive and added a lot of value to the work!

- I didn't see a statement about ethical approval, is this available and could this be added?

This section has now been added.

Results:
- Quantitative results were clearly explained. When describing the results of Table 2, selected results were mentioned, but these were based on small numbers and are not statistically significant, which should be mentioned.

Thank you we have clarified this point.

- Could you please clarify how much missing data there was and how this was handled?

We have clarified this point in the manuscript. We did not run any additional tests to assess for the effect of missing data on the stated results.

Discussion:

- As mentioned earlier, additional information about PNC experiences pre-pandemic would be useful (I understand this data was not collected in this study, but experiences from the literature).

Thank you. We have added some additional description and discussion of the PNC landscape pre-COVID.

- It would be helpful to add a section in describing the potential for confounding in this study and either the implications of this, or how future work could address potential confounding.

Thank you. We have added some additional commentary in relation to this point.

- How does this study sample relate to the population in the wider study? Is it possible to discuss the generalizability of this study to the wider population in the ICS?

We have clarified this point in the discussion and referred to the previous paper that includes some data describing the demographic representativeness of the sample.

- These findings are consistent with papers published describing experiences of accessing contraceptive care during lockdowns, and some of this literature could be included/referenced.

- It might be useful to refer the reader to the timeline of lockdowns and a reminder of the social restrictions in place. For example, could the social restrictions during the lockdown period have been the reason why male partners were less involved in PNC discussions? (This broader context may be useful to help interpretation).

Thank you. We have updated the discussion to include some additional consideration of these points.

Many thanks for the opportunity to review this paper and I hope these comments are useful to the authors.

Reviewer: 2

Dr. Jayalakshmy Ramakrishnan, JIPMER

Comments to the Author:

The study objective was very pertinent, and it was highlighted in the manuscript. However, the data collection through digital medium was a limitation in this study. it was mentioned as well in the manuscript. The qualitative could have been more elaborate, it was not addressing several health system limitations in the study. Another stakeholder meeting could have to added to address the health system's perspective of the problem.

Thank you for this feedback. We did collect qualitative data from healthcare professionals in online focus groups, but it is our intention to share these accounts in a separate paper. The discussions with healthcare professionals were not explicitly in relation to pandemic experiences.

Thank you again for considering our paper for publication.

VERSION 2 - REVIEW

**Reviewer**	**1**
**Name**	**Foley, Kimberley**
**Affiliation**	**Imperial College London, Primary Care and Public Health**
**Date**	**19-May-2025**
**COI**	

Thank you for addressing my previous comments, I have no further suggestions.

My only further thought is that there is a study I previously contributed to that did look at contraceptive use across the lockdown period. Although this study was not focused on PNC only, I still wondered if it might be relevant. If interested, the study is: https://doi.org/10.3399/BJGPO.2021.0218
